# Benign ovarian lesions with restricted diffusion

**DOI:** 10.1590/0100-3984.2018.0078

**Published:** 2019

**Authors:** Lisa Agostinho, Mariana Horta, João Cunha Salvador, Teresa Margarida Cunha

**Affiliations:** 1 Hospital Beatriz Angelo - Radiologia, Loures, Portugal.; 2 Instituto Português de Oncologia de Lisboa Francisco Gentil - Radiologia, Lisboa, Portugal.

**Keywords:** Diffusion magnetic resonance imaging, Magnetic resonance imaging, Pelvic neoplasms, Ovary, Imagem de difusão por ressonância magnética, Ressonância magnética, Neoplasias pélvicas, Ovário

## Abstract

Developments in magnetic resonance imaging have expanded its role in the assessment of the female pelvis, including the diagnosis of ovarian lesions. In this setting, diffusion-weighted imaging has proven its diagnostic value, which is particularly important in differentiating between benign and malignant ovarian tumors. In general, the latter show restricted diffusion, whereas the former do not. Exceptions include teratomas, endometriomas, hemorrhagic cysts, ovarian abscesses, ovarian infarction, and some benign stromal tumors. The aim of this review is to draw attention to benign ovarian lesions with restricted diffusion, with a special focus on diffusion-weighted imaging pearls and pitfalls.

## Abstract (pt)

Os recentes desenvolvimentos nas técnicas de ressonância magnética aumentaram o seu papel na avaliação da pelve em mulheres, incluindo o diagnóstico de lesões ovarianas. A imagem ponderada em difusão comprovou ter um valor diagnóstico indiscutível, fato particularmente importante na diferenciação entre tumores ovarianos benignos e malignos. Em geral, os últimos têm restrição na difusão, enquanto os primeiros não. As exceções incluem teratomas, endometriomas, cistos hemorrágicos, abcessos ovarianos, enfarte ovariano e alguns tumores benignos do estroma ovariano. Esta revisão tem como objetivo expor lesões ovarianas benignas com restrição na difusão, com especial enfoque nas pérolas e armadilhas da imagem ponderada em difusão.

## INTRODUCTION

In ovarian disorders, conventional morphological evaluation in T1-weighted (T1W) and T2-weighted (T2W) magnetic resonance imaging (MRI) sequences is essential. However, diffusion-weighted imaging (DWI) has undeniable diagnostic value, providing excellent tissue contrast based on the molecular diffusion of water^(^^1^^)^. This fact is particularly relevant in differentiating between benign and malignant ovarian tumors^(^^2^^)^. In general, the latter show restricted diffusion, whereas the former do not. Nevertheless, there are some exceptions. The restricted diffusion of water molecules is proportional to the following: expansion of the intracellular compartment, as in cytotoxic edema; increased cell membrane density due to hypercellularity; increased fluid viscosity; and increased tortuosity of the extracellular space^(^^3^^)^. In fact, restricted diffusion may be observed in normal tissue, in solid non-malignant lesions with high cellular density, or in cystic non-malignant lesions with high viscosity^(^^3^^)^. To avoid diagnostic pitfalls, DWI should always be evaluated in conjunction with the apparent diffusion coefficient (ADC) maps and correlated with conventional anatomic MRI scans (T1W and T2W sequences).

## DWI

### DWI basics

DWI is a functional MRI technique, performed without contrast administration, that provides information about the intracellular, transcellular, and extracellular Brownian motion of water molecules in a tissue (true diffusion), as well as the microcirculation (perfusion), which can be evaluated separately^(^^1^^)^. In tissues with increased cellularity and a resultant increase in cell membrane surface area, there is a decrease in Brownian motion (i.e., restricted diffusion), whereas there is an increase in Brownian motion (i.e., free diffusion) in tissues with decreased cellularity. The ADC, which represents a combination of true diffusion and perfusion, is a numerical value assigned to the degree of such motion in a tissue and has been used as a marker of cellularity.

Free diffusion, qualitatively visible as progressive signal loss on DWI obtained with increasing b values and quantitatively measured as a region with a high ADC, can be seen in tissues with low cellularity^(^^4^^)^. In contrast, restricted diffusion, qualitatively visible as high signal intensity on DWI obtained with increasing b values and quantitatively measured as a region with a low ADC, is associated with hypercellularity and has been used as an indicator of potential malignancy^(^^4^^)^.

In DWI, the b values represent different diffusion-sensitizing gradient strengths and are expressed as s/mm^2^ (time/area). Because the ADC is the slope of exponential decrease in signal intensities between DWI sequences with different b values, DWI with ADC mapping is performed with at least two different b values. At lower b values, the perfusion-related contribution is higher, which can affect the ADC, and the image mirrors a heavily T2W fat-suppressed sequence. The predominant contributor changes from perfusion to diffusion at b values in the 100-300 s/mm^2^ range^(^^1^^)^. At higher b values (> 1000 s/mm^2^), there can be a progressive decrease in the signal-to-noise ratio^(^^5^^)^.

The ADC is expressed as a numerical value × 10^−3^ mm^2^/s and represents a quantitative metric. It quantifies the progressive loss of signal in a tissue of interest visible on DWI performed with successively increasing b values. A region of interest can be drawn by the radiologist in the tissue of interest to obtain a numeric value^(^^4^^)^. The region of interest results in automatically generated ADCs, presented as minimum, mean, and maximum values or as a mean value.

In pelvic examinations, DWI is most commonly acquired in the axial plane. However, obtaining sequences with the same orientation as that of T2W images allows fusion imaging and optimizes anatomic correlation^(^^6^^)^.

### Pitfalls in DWI interpretation

One should bear in mind some pitfalls when interpreting DWI sequences. In general, high-cellularity tumors demonstrate restricted diffusion, whereas normal tissue does not. However, benign tissue components, such as blood, fat, necrosis and pus, may also show restricted diffusion^(^^6^^)^. As a result, DWI characteristics of benign and malignant tumors, including those of the ovary, may overlap^(^^7^^)^. It is crucial to correlate DWI and ADC data with morphological characteristics to minimize the probability of misinterpretation.

It is also important to keep in mind the fact that DWI is based on T2W images, causing tissues with long T2 relaxation time, such as simple cysts, to show high signal intensity-the so-called T2-shine-through effect. However, an ovarian mass with a hypointense signal on DWI, the ADC map, and T2W images-the so-called T2-blackout effect-is most likely a benign mass^(^^2^^)^, making DWI particularly suited to excluding the possibility of malignancy.

## THE OVARY ON MRI

### The normal ovary

The ovaries are usually ovoid in shape and average 10 cm^3^ in volume^(^^8^^)^. The zonal anatomy is best appreciated on T2W images, the normal medulla showing increased signal intensity relative to the cortex, reflecting the greater amount of loosely packed stroma and blood vessels, and diminished cellularity of the medulla^(^^8^^)^. In women of reproductive age, there are multiple follicles in the cortex, appearing as thin-walled cystic structures, the fluid content of which results in high signal intensity on T2W images and low signal intensity on T1W images^(^^8^^)^. On T2W images, the corpus luteum demonstrates wall thickening, with low to intermediate signal intensity, that corresponds to a layer of luteinized theca cells^(^^8^^)^.

In women of reproductive age, the normal ovary may have high enough signal intensity to be identified on DWI sequences from zero to high b values, especially during the luteal phase. Ovarian follicles may show high signal intensity on all DWI sequences (even at high b values), with concomitant high ADCs^(^^9^^)^.

### Benign ovarian lesions with restricted diffusion

#### Mature cystic teratoma

Mature cystic teratomas are the most common ovarian tumors in women under 45 years of age^(^^10^^)^. They are composed of mature tissue from at least two of the three germ cell layers (endoderm, mesoderm, and ectoderm); in 88% of cases, they are filled with sebaceous material, which is liquid at body temperature, and are lined with keratinized squamous epithelium^(^^11^^)^. They grow slowly, at an average rate of 1.8 mm each year, prompting some researchers to advocate nonsurgical management of smaller (< 6 cm) tumors^(^^12^^)^. In 10% of cases, the tumors are bilateral^(^^13^^)^. Mature cystic teratomas contain hair follicles, skin glands, muscle, and other tissues. There is usually a raised protuberance projecting into the cyst cavity, known as a Rokitansky nodule^(^^13^^)^. Ectodermal tissue (skin and neural derivatives) is invariably present, as are mesodermal tissue (fat, bone, cartilage, and muscle) in over 90% of cases and endodermal tissue (e.g., gastrointestinal epithelium, bronchial epithelium, and thyroid tissue) in most cases^(^^13^^)^. Although it is one of the most relevant features, adipose tissue is present in only 67-75% of cases^(^^13^^)^.

Mature cystic teratomas have typical imaging features on MRI (Table 1). Their adipose content displays a signal that is hyperintense on T1W images and becomes hypointense after selective fat saturation. This characteristic is sufficient to establish the diagnosis of a ovarian teratoma^(^^13^^)^.

**Table 1:** Findings in benign ovarian lesions with restricted diffusion.

Lesion	Signal intensity		b = 1000 s/mm^2^	ADC
	T2	T1		
Teratoma	Variable, heterogeneous	High, but low on T1 with FS	High	Low
Hemorrhagic cyst	High to intermediate; low in chronic hemorrhage	High	High	Low
Endometrioma	Low to intermediate	High	High	Low
Ovarian torsion*	High	High	High	Low
Tubo-ovarian abscess	Variable, heterogeneous	Variable	High	Low
Functioning thecoma and cellular	Intermediate to low; hyperintense in areas of edema or cystic	Low	High	Low
fibroma	degeneration			

* With hemorrhagic infarction. FS, fat saturation.

**Figure 1:**
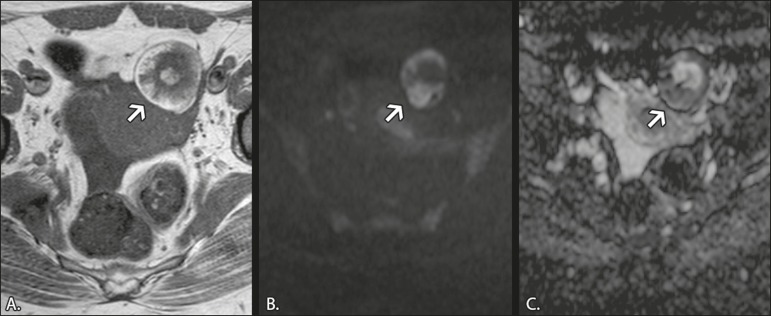
MRI of a 32-year-old female patient with a left mature cystic teratoma. **A:** Axial T1W image showing a cystic lesion with areas of high signal intensity in which the signal became hypointense after fat saturation (not represented). **B:** Axial DWI at b = 1000 s/mm^2^, showing areas of high signal intensity within the lesion. **C:** ADC map showing low ADCs within the lesion.

It has been suggested that the abnormal signal intensity displayed on DWI by most mature cystic teratomas is caused by the presence of keratin, a protein originating from the cytoskeletal structure of the epidermis^(^^7^^)^. Other sites showing abnormal signal intensity include Rokitansky nodules and fat globules^(^^7^^)^. The restricted Brownian movement of water molecules within the keratinous substance results in a high signal intensity on DWI and low ADCs (Figure 1). In fact, DWI can facilitate the diagnosis of mature cystic teratomas, especially those containing a small amount of fat that is not detectable by conventional MRI^(^^14^^,^^15^^)^.

Mature cystic teratomas undergo malignant transformation in 1-2% of the cases^(^^13^^)^. Malignant transformation tends to occur in older women (between 60 and 70 years of age), squamous cell carcinoma being the most common histological type. In such cases, they can show restricted diffusion in their solid component, due to hypercellularity^(^^10^^)^. The morphological correlation is mandatory, because malignant mural nodules tend to show intermediate signal intensity on T2W images and enhancement after gadolinium administration^(^^15^^)^.

#### Hemorrhagic cyst and endometrioma

When an ovarian follicle enlarges during the menstrual cycle but does not rupture for ovulation, a follicular cyst, typically measuring between 3 cm and 6 cm, may develop. In some cases, these cysts undergo hemorrhage, becoming hemorrhagic cysts. On MRI, a hemorrhagic cyst usually has high signal intensity on T1W images and intermediate to high signal intensity on T2W images (Table 1). There is smooth enhancement on the cyst wall, without vegetations or nodularity^(^^6^^)^. Because of their blood content, hemorrhagic cysts may display restricted diffusion^(^^6^^)^.

Endometriomas, also known as endometriotic cysts, are benign cysts that constitute an ovarian manifestation of endometriosis. On MRI, endometriomas typically appear as cystic lesions, typically multiple or bilateral, with thick walls^(^^16^^)^. They show high signal intensity on T1W images, with or without selective fat suppression, and relatively low signal intensity on T2W images-the shading sign^(^^17^^)^. However, this sign is not exclusive to endometriomas, given the fact that T2 shading can also be observed in hemorrhagic cysts and other benign or malignant non-endometrioid adnexal tumors^(^^18^^)^, leading to diagnostic difficulties.

**Figure 2:**
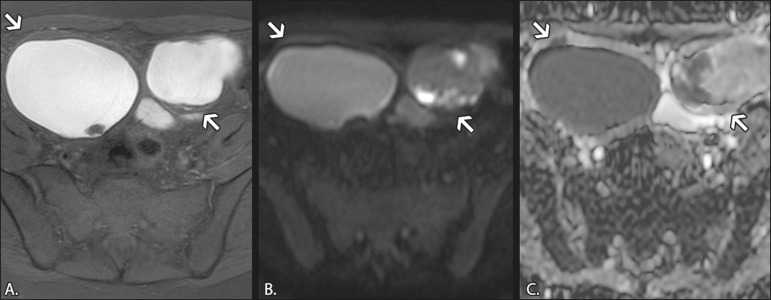
MRI of a 41-year-old female patient with endometriomas. **A:** Axial T1W image with fat suppression, showing bilateral cystic lesions with high signal intensity. **B:** Axial DWI at b = 1000 s/mm^2^, showing areas of high signal intensity within the lesions. **C:** ADC map showing low ADCs within the lesions.

Because endometriomas contain blood and some hemosiderin, they show T1 shortening, high signal intensity on DWI, and lower ADCs^(^^7^^)^, as depicted in Figure 2. The presence of restricted diffusion in endometriomas may hamper the detection of malignant transformation, making the correlation with other sequences mandatory. Solid nodules showing intermediate signal intensity on T2W images, peritoneal metastases, thickened septa (> 3 mm), or contrast enhancement are likely to be malignant^(^^19^^)^. Clear cell and endometrioid carcinomas are the most common malignant tumors associated with endometriomas.

DWI and ADC mapping may play roles in differentiating between hemorrhagic cysts and endometrioma. In a recent study, including 24 benign cystic hemorrhagic adnexal lesions, Balaban et al.^(^^20^^)^ reported that ADCs were significantly lower in endometriomas than in hemorrhagic cysts, a fact that can be used to differentiate between the two conditions. There are, however, more reliable ways of making this distinction, in particular the presence of the T2 dark spot sign, which has high specificity for chronic hemorrhage^(^^16^^)^.

#### Ovarian torsion

Ovarian torsion usually presents as acute severe pelvic pain and is caused by partial or complete rotation of the ovarian vascular pedicle. Predisposing factors include an underlying ovarian tumor (especially one > 6 cm), hypermobile adnexa, and elongated fallopian tubes^(^^21^^)^. Ovarian torsion most commonly affects women under 30 years of age^(^^21^^)^.

In cases of ovarian torsion, venous blood flow is initially compromised, causing edema and swelling. Later, the dual arterial blood supply is also compromised, leading to hemorrhagic infarction^(^^22^^)^, which in turn results in irreversible loss of the ovary.

The most common characteristics of the lesion in adults are an enlarged ovary with areas of signal hyperintensity on T1W and T2W images (Table 1), due to hemorrhage and edema, respectively. Peripheral follicles can also be observed^(^^23^^)^. In the late phases, gangrenous necrosis develops.

To date, there have been no studies demonstrating that the use of DWI adds value in the diagnosis of ovarian torsion. However, DWI has been shown to be beneficial in identifying hemorrhagic infarction^(^^24^^)^: low ADCs are more common in torsed ovaries with hemorrhagic infarction than in those without.

#### Tubo-ovarian abscess

In the majority of cases, tubo-ovarian abscesses result from pelvic inflammatory disease, being more common in women of reproductive age^(^^25^^)^. Tubo-ovarian abscesses are thick-walled, multilocular adnexal masses. They may contain septa, pus, gas, or fluid, with or without fluid-debris levels^(^^25^^)^. The content usually displays a heterogeneously intermediate signal on T2W images (Table 1). Areas of necrosis or loculated fluid collections may resemble serous fluid but can also be proteinaceous or hemorrhagic with T1 shortening^(^^26^^)^. Tubo-ovarian abscesses are surrounded by thick, markedly enhancing outer borders.

**Figure 3:**
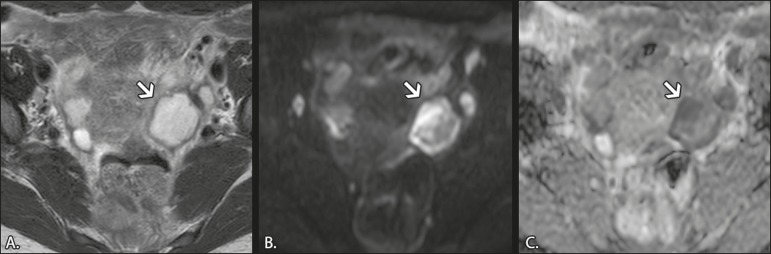
MRI of a 39-year-old female patient with a left tubo-ovarian abscess. **A:** Axial T2W image showing a cystic lesion with heterogeneous high-signalintensity content. **B:** Axial DWI at b = 1000 s/mm^2^, showing areas of high signal intensity within the lesion. **C:** ADC map showing low ADCs within the lesion.

Tubo-ovarian abscesses comprise viscous proteinaceous fluid containing bacteria, inflammatory cells, cellular debris, and necrotic tissue. When the content is more viscous, the signal intensity is higher on DWI and lower on the ADC map^(^^27^^)^, as shown in Figure 3. Depending on the viscosity of the pus, the abscess contents may show heterogeneous restricted diffusion. Chronic abscesses, abscesses smaller than 1 cm in diameter, and abscesses in patients on antibiotic therapy may not show restricted diffusion^(^^28^^)^.

DWI also plays a role in making a distinction between tubo-ovarian abscesses and cystic or necrotic neoplasms. Neoplasms typically show restricted diffusion at the periphery, where the cell density is higher, whereas abscesses show central restricted diffusion^(^^24^^)^.

#### Fibroma and thecoma

Fibromas are the most common solid ovarian tumors and account for 4% of all ovarian neoplasms^(^^29^^)^. Fibromas are benign tumors that can present at any age, although the mean age of occurrence is in the late forties. Fibromas that are highly cellular are classified as either cellular fibromas or fibrosarcomas. Cellular fibromas constitute 10% of all ovarian fibromas and have low malignant potential, whereas fibrosarcomas are malignant tumors^(^^29^^,^^30^^)^.

On MRI, fibromas usually present low signal intensity on T1W images, low signal intensity on T2W images, and weak, delayed enhancement after contrast administration. In the majority of these tumors, T2-shortening effects, caused by the abundant collagen contents and the decreased extracellular fluid, result in a signal decrease on T2W images^(^^31^^)^. The pathology examination can reveal edema and cystic degeneration, which explain the varying degree of intermediate to high signal intensity portions on T2W images, which may mimic malignancy, in some cases^(^^32^^)^.

Thecomas account for 0.5-1.0% of all primary ovarian tumors and are most likely to occur in postmenopausal women^(^^29^^)^. In most cases, they exhibit estrogenic activity^(^^33^^)^. With rare exceptions, they are considered benign neoplasms^(^^29^^)^.

**Figure 4:**
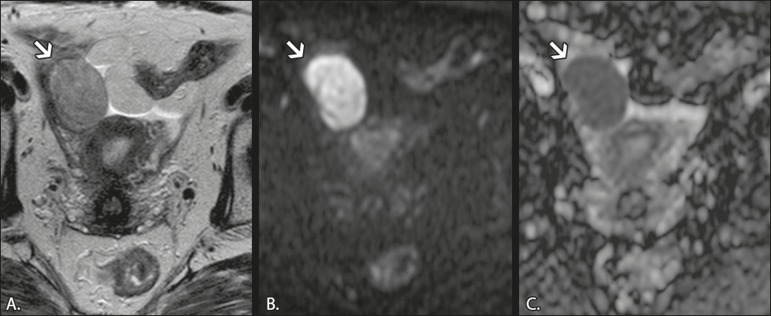
MRI of a 67-year-old female patient with thecoma. **A:** Axial T2W image showing a solid lesion with intermediate signal intensity. **B:** Axial DWI at b = 1000 s/mm^2^, showing high signal intensity within the lesion. **C:** ADC map showing low ADCs within the lesion.

On MRI, when compared with predominantly fibrous tumors, pure thecomas tend to exhibit higher signal intensity on T2W images and more avid contrast enhancement, a major pitfall in the diagnosis of malignant ovarian tumors of the ovary^(^^30^^)^. The accurate diagnosis of these tumors and their differentiation from malignant tumors is critical for correct patient management. DWI, in combination with ADC mapping, may play a role in making that distinction. According to the most recent European Society of Urogenital Radiology guidelines for indeterminate adnexal masses, a solid lesion that exhibits low signal intensity on T2W images and on DWI with high b values is highly likely to be benign^(^^2^^,^^34^^)^. However, isolated ADC measurements in the solid component have not been found to contribute to differentiating between benign and malignant adnexal masses, possibly due to the lower mean ADCs in benign fibrous tumors associated with dense fibrous stromal proliferation. In such tumors, no signal increase is observed on DWI, despite their low ADCs, probably as a consequence of the T2 blackout effect^(^^2^^)^. Nevertheless, some fibromas and thecomas in this group may show restricted diffusion. For instance, functioning thecomas and cellular fibromas may show high signal intensity on DWI and relatively low ADCs (Figure 4), due to their relatively high cellularity^(^^35^^-^^37^^)^.

## CONCLUSION

In ovarian disorders, conventional morphological evaluation with T1W and T2W images is essential. However, DWI has experienced a rise in popularity, due to its undeniable diagnostic value, particularly in differentiating between benign and malignant tumors. It is advisable to use DWI as a complementary sequence, whenever possible, because it can provide excellent tissue contrast based on the molecular diffusion of water within tumors. As a general rule, malignant tumors present restricted diffusion, whereas benign tumors do not. Nevertheless, radiologists should be aware of some lesions that constitute exceptions to that rule: teratomas, endometriomas, hemorrhagic cysts, ovarian abscesses, ovarian infarction, and some benign sex cord-stromal tumors.
